# Podocyte number and glomerulosclerosis indices are associated with the response to therapy for primary focal segmental glomerulosclerosis

**DOI:** 10.3389/fmed.2024.1343161

**Published:** 2024-03-06

**Authors:** Natasha de Zoysa, Kotaro Haruhara, David J. Nikolic-Paterson, Peter G. Kerr, Jonathan Ling, Sarah E. Gazzard, Victor G. Puelles, John F. Bertram, Luise A. Cullen-McEwen

**Affiliations:** ^1^Department of Anatomy and Developmental Biology, Monash Biomedicine Discovery Institute, Clayton, VIC, Australia; ^2^Division of Nephrology and Hypertension, Jikei University School of Medicine, Tokyo, Japan; ^3^Department of Nephrology, Monash Medical Centre, Clayton, VIC, Australia; ^4^Monash University Department of Medicine, Monash Medical Centre, Clayton, VIC, Australia; ^5^III. Department of Medicine, University Medical Center Hamburg-Eppendorf, Hamburg, Germany; ^6^Department of Clinical Medicine, Aarhus University, Aarhus, Denmark; ^7^Department of Pathology, Aarhus University Hospital, Aarhus, Denmark; ^8^ARC Training Centre for Cell and Tissue Engineering Technologies, Melbourne, VIC, Australia; ^9^ARC Training Centre for Cell and Tissue Engineering Technologies, Brisbane, QLD, Australia

**Keywords:** kidney, FSGS, podocyte, therapy, podometrics

## Abstract

Corticosteroid therapy, often in combination with inhibition of the renin-angiotensin system, is first-line therapy for primary focal and segmental glomerulosclerosis (FSGS) with nephrotic-range proteinuria. However, the response to treatment is variable, and therefore new approaches to indicate the response to therapy are required. Podocyte depletion is a hallmark of early FSGS, and here we investigated whether podocyte number, density and/or size in diagnostic biopsies and/or the degree of glomerulosclerosis could indicate the clinical response to first-line therapy. In this retrospective single center cohort study, 19 participants (13 responders, 6 non-responders) were included. Biopsies obtained at diagnosis were prepared for analysis of podocyte number, density and size using design-based stereology. Renal function and proteinuria were assessed 6 months after therapy commenced. Responders and non-responders had similar levels of proteinuria at the time of biopsy and similar kidney function. Patients who did not respond to treatment at 6 months had a significantly higher percentage of glomeruli with global sclerosis than responders (*p* < 0.05) and glomerulosclerotic index (*p* < 0.05). Podocyte number per glomerulus in responders was 279 (203–507; median, IQR), 50% greater than that of non-responders (186, 118–310; *p <* 0.05). These findings suggest that primary FSGS patients with higher podocyte number per glomerulus and less advanced glomerulosclerosis are more likely to respond to first-line therapy at 6 months. A podocyte number less than approximately 216 per glomerulus, a GSI greater than 1 and percentage global sclerosis greater than approximately 20% are associated with a lack of response to therapy. Larger, prospective studies are warranted to confirm whether these parameters may help inform therapeutic decision making at the time of diagnosis of primary FSGS.

## Introduction

Focal segmental glomerulosclerosis (FSGS) is characterized by sclerosis in some but not all glomeruli and only in parts of individual glomeruli. The incidence of FSGS ranges from 0.2 to 1.8/100,000 population per year (1), which continues to increase in diverse populations (2–5). Primary FSGS refers to an idiopathic form of disease where the mechanisms of pathogenesis are poorly understood. However, it is generally accepted that podocyte loss is a cause of segmental lesion formation, suggesting that podocyte injury is a key event in the development of FSGS (6, 7).

Standard first-line therapy for patients with primary FSGS with nephrotic-range proteinuria involves corticosteroids, often also employed with blockade of the renin angiotensin system (RAS) (8, 9). Complete remission is defined as proteinuria ≤0.3 g/24 h, or protein to creatinine ratio of <30 mg/mmol, stable serum creatinine and serum albumin>35 g/L, whilst partial remission is defined as a > 50% reduction in peak proteinuria and/or to sub-nephrotic levels (0.3–3.5 g/24 h or 30–350 mg/mmol total protein to creatinine ratio) (9–11). Unfortunately, response to first-line therapy is variable and unpredictable, with 40–60% of patients failing to achieve remission or having relapsing proteinuria (defined as proteinuria >3.5 g/d after complete remission was achieved) (8, 12), both of which are largely attributed to steroid resistance (13–18). There is therefore significant clinical value in developing approaches to indicate the response to first-line therapy in primary FSGS, particularly to avoid the severe multi-organ side effects of corticosteroids (19–21) in patients who are not likely to respond to steroid therapy (22).

Murine and human podocytes express corticosteroid (23–25) and angiotensin receptors (26) suggesting that standard first-line therapy for primary FSGS may have direct action on podocytes that are independent of their systemic effects. *In vivo* and *in vitro* studies have provided strong evidence that steroids and RAS blockade have direct therapeutic effects on podocytes, including enhanced podocyte survival, resulting in the preservation of podocyte number (24, 27–29). Findings from animal studies indicate that the degree of podocyte depletion is associated with the degree of resulting pathology (7). Taken together, these findings suggest that the degree of podocyte depletion at the time of primary FSGS diagnosis may be associated with the subsequent response to therapy.

Using diagnostic biopsies from patients with primary FSGS, this study aimed to determine if podocyte number, density and/or size and indices of glomerulosclerosis can indicate the clinical response to first-line therapy in patients with primary FSGS. The findings indicate that podocyte number per glomerulus as well as the degree of glomerulosclerosis at the time of diagnosis may aid in deciding therapy regimens.

## Materials and methods

### Study overview and patient inclusion/exclusion criteria

**Figure 1:**
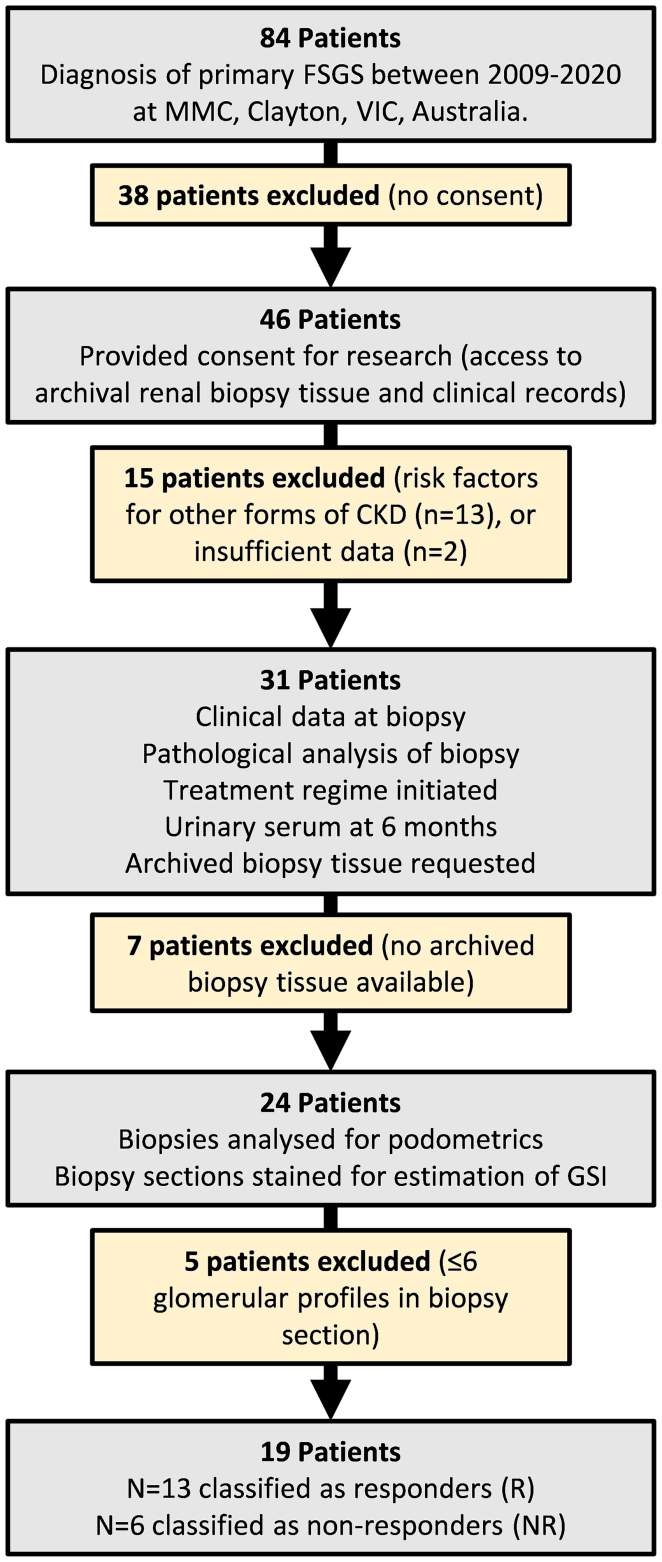
Flowchart of study design and patient inclusion/exclusion criteria. 84 patients were initially considered for the study. 65 patients were excluded. The remaining 19 patients were grouped into responders and non-responders based on their urinary/serum data 6 months after commencing treatment.

A retrospective single center cohort study was undertaken between 2018 and 2020 at Monash Medical Centre (MMC), Monash Health in Melbourne, Australia. The study protocol was approved by the Monash University Human Research Ethics Committee (14344). The study used archival kidney biopsy tissue obtained from patients who required renal biopsy to confirm diagnosis of primary FSGS between 2009 and 2020. While 84 patients were considered for the study, 65 patients were excluded as summarized in Figure 1. 19 patients were included. Patients were considered treatment responders (R) if they entered remission as classified by the KDIGO guidelines (11), that is when proteinuria was <3.5 g/d (reported or calculated), total protein to creatinine ratio < 350 mg/mmol, and stable serum creatinine (11). Patients were considered treatment non-responders (NR) if they did not satisfy the above criteria. Thirteen patients were classified as responders and six were classified as non-responders.

All participants provided written informed consent for their biopsy specimen to be used for research and access to the following clinical data: age at time of biopsy, urinary/serum data at time of biopsy, pathology analysis of biopsy, treatment regime initiated, and urinary/serum data 6 months after diagnosis.

### Diagnosis of primary FSGS

Primary FSGS at MMC is diagnosed by clinical presentation and renal biopsy showing segmental increase of the mesangium, segmental sclerosis and podocyte foot process effacement with electron microscopy. Biopsies were obtained if a patient presented with one, or a combination of idiopathic proteinuria (>1.0 g/24 h), abnormal renal function (<90 mL/min/1.73 m^2^) or hematuria. Biopsy tissue was divided and fixed in 10% formalin, embedded in paraffin, sectioned at 1 μm and stained with hematoxylin and eosin, PAS, silver methenamine/Masson trichrome or orcein/Masson trichrome. Finally, clinical notes were examined to ensure no causes of secondary FSGS were noted at the time of biopsy (30).

### Clinical data

Clinical data for patients with biopsy-confirmed primary FSGS was collected by accessing pathology results and scanned medical records. Information collected included age at time of biopsy, sex, ethnicity, urinary/serum data at time of biopsy, descriptive pathology analysis of biopsy, treatment regime initiated, and urinary/serum data 6 months after diagnosis. Self-reported place of birth was used as a proxy of ethnicity; where Caucasians were defined as patients born in Australia (*n* = 15) or Greece (*n* = 1), and Asians were defined as patients born in Sri Lanka (*n* = 1), India (*n* = 1) or Singapore (*n* = 1). Medical history at time of biopsy was examined for infection, malignancy, associated medical issues such as diabetes, and the presence of any genetic testing as these are causes of secondary FSGS (as above).

#### Proteinuria

Proteinuria was reported as either 24-h protein excretion, urinary total protein relative to creatinine (UTP/Cre), individual urinary total protein and creatinine measurements, or albumin relative to creatinine (ACR). Proteinuria as 24-h protein excretion is commonly considered the gold standard. However, in this retrospective study, 24-h protein excretion was not available for three patients at the time of biopsy (2R and 1NR), or 12 patients 6 months after treatment commenced (10R and 2NR). For the three patients at biopsy and 10 of the 12 patients at 6 months, individual urinary total protein and creatinine values were used to calculate an estimate of protein excretion in grams/day for consistency using MediCalc^®^ Medical Calculator System designed by ScyMed^®^ (31–33). Individual protein and creatinine values were not reported 6 months after treatment commenced in two patients (1R and 1NR). Classification of these two patients was based on reported ACR [equivalent to <0.5 g/day (34); 1R] or increased serum creatinine from baseline (1NR) (11).

#### Estimated glomerular filtration rate (eGFR)

Various equations were used to calculate eGFR, incorporating variables such as serum creatinine, age, weight, race and sex. To achieve uniformity, eGFR at time of biopsy was recalculated for all patients using the Chronic Kidney Disease Epidemiology Collaboration (CKD-EPI) equation (35), taking into account reported serum creatinine, age and sex as recommended by clinical practice guidelines (36, 37). eGFR was also calculated for patients 6 months after their treatment was initiated. However, three patients did not have serum creatinine measured at 6 months. Each group had one patient <18 years of age, which is significant as the current CKD-EPI formula cannot be used on pediatric patients (38). Therefore, these patients did not have eGFR calculated at time of biopsy or after 6 months of treatment.

### Podocyte immunofluorescent staining and imaging

**Figure 2:**
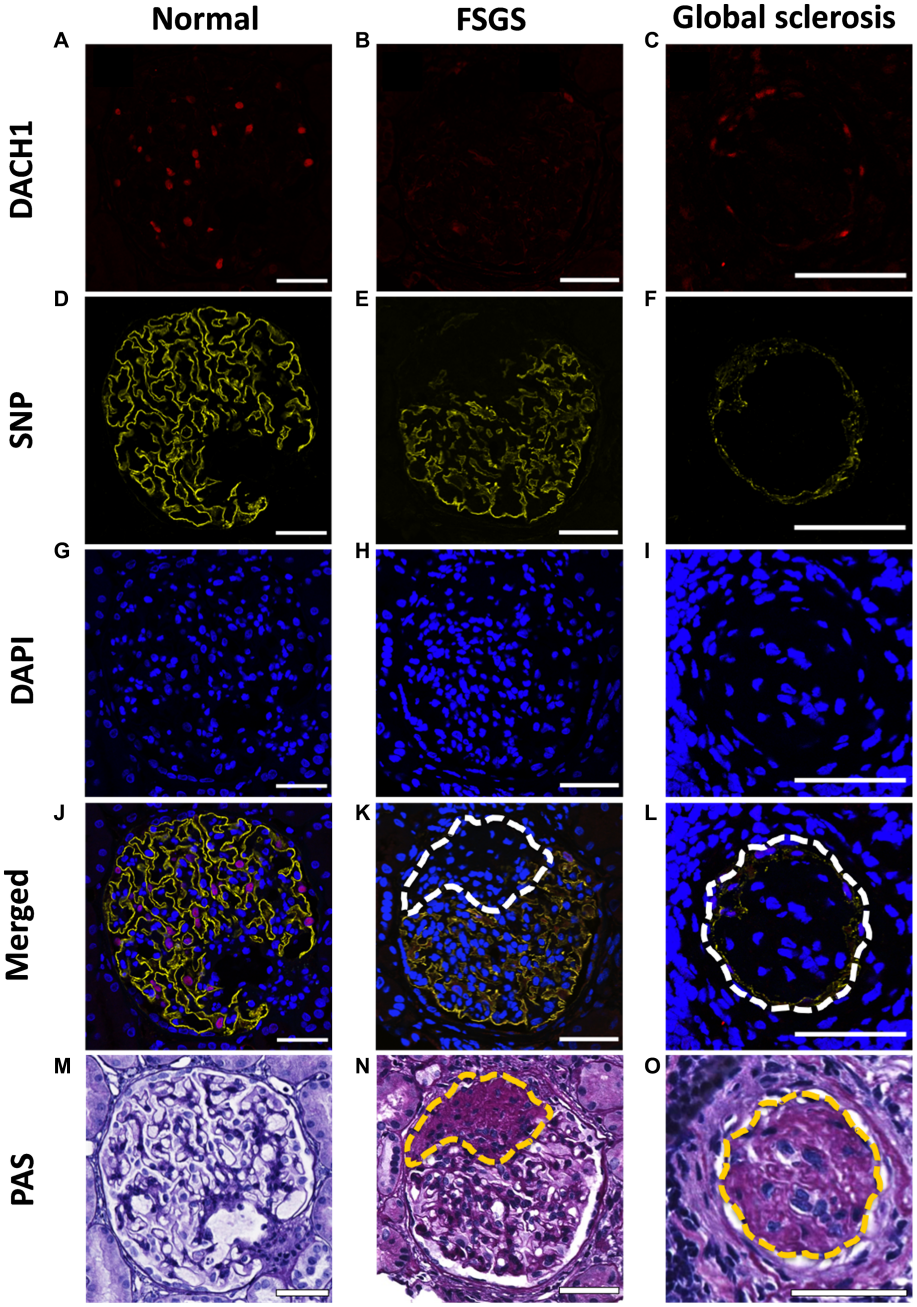
Immunofluorescent and PAS staining of biopsies. Representative images of a normal glomerulus, glomerulus with FSGS and glomerulus with global sclerosis immunofluorescently stained for DACH1 (podocyte nuclei; red; **A–C**), synaptopodin (SNP; podocyte cytoplasm; yellow; **D–F**) and DAPI (all nuclei; blue; **G–I**). Merged images show podocyte nuclei as pink where DACH1 and DAPI merge **(J–L)**. Following immunofluorescence imaging, sections were re-stained with PAS **(M–O)**. Dashed lines indicate sclerotic lesions, which in the immunofluorescent images lack DACH1 and SNP staining. Scale bars = 50 μm.

Multiple strategies have been used to estimate podocyte number and density with many different marker combinations, including transducin-like enhancer of split 4 (TLE4) (39), DACH1 (40), p57 (41) and WT1 (42). After testing for different antibody combinations in our tissues, we proceeded with DACH1 (to mark podocyte nuclei; Figures 2A–C) and Synaptopodin (to mark podocyte cytoplasm; Figures 2D–F) and DAPI (to mark all nuclei; Figures 2G–I). Following de-waxing and dehydration, sections were subject to antigen retrieval for 2 h at 92°C in a buffer of pH 9. Sections were blocked with 1% bovine serum albumin in 0.05% PBS/Tween-20 (P1379; Sigma Aldrich) solution for 1 h at room temperature (RT). Sections were then immunostained using polyclonal rabbit anti-DACH1 (daschund1; 1:1000; 10914-1-AP; Proteintech) and monoclonal mouse anti-synaptopodin (1:500; SC-515842; Santa Cruz Biotechnology) antibodies to label podocyte nuclei (Figures 2A–C) and podocyte cytoplasm (Figures 2D–F), respectively. Primary antibodies were diluted in a solution of 1% bovine serum albumin (BSA; A7906; Sigma-Aldrich) and 0.05% PBS-Tween-20, and incubated at 4°C overnight. After a wash step, primary antibodies were fluorescently labeled with Alexa Fluor 633-conjugated goat anti-rabbit IgG antibody (1:1000; A21070; Invitrogen) and Alexa Fluor 568-conjugated goat anti-mouse IgG antibody (1:1000; A11004; Invitrogen) for 2 h at RT. Secondary antibodies were diluted in a solution of 10% human serum (H4522; Sigma Aldrich) and PBS/Tween-20. An autofluorescence quenching kit with DAPI (4′,6′-diamidino-2-phenylindole) was then used to identify all nuclei (SP-8500; Vector laboratories; Figures 2G–I). Images were obtained using a Leica SP5 laser confocal microscope (Leica Microsystems) using a x40 oil immersion objective lens (1.25 numerical aperture) and x1.6 set zoom. All images were taken with eight line-averages and stored in a 1,024 × 1,024-pixel frame.

All glomerular profiles present on a biopsy section were imaged (14 ± 7 glomeruli per section; mean ± SD), inclusive of normal glomeruli (Figures 2J,M) and those with varying levels of sclerosis (Figures 2K,N) and global sclerosis (Figures 2L,O). Patients whose biopsies contained ≤6 glomeruli in a section were excluded. PAS images were used to confirm the number of glomeruli present per section, irrespective of level of sclerosis. If any section had one or more glomeruli that were not imaged in immunofluorescence (due to global sclerosis), a serial section of the biopsy was re-stained and re-imaged. This double staining validation allowed glomeruli with varying levels of sclerosis to be included in both podometric and pathology analyses.

#### Glomerulosclerotic index (GSI)

Sections immunofluorescently stained for podometric analysis were re-stained with PAS to quantify GSI in the same glomeruli used for podometric assessment (43, 44) and to confirm glomerular profile number per section. The degree of sclerosis in each glomerular profile was subjectively scored from 0 to 4: score 0, no sclerosis; score 1, sclerosis occupies 1–25% of the glomerular area; score 2, sclerosis occupies 26–50% of the glomerular area; score 3, sclerosis occupies 51–75% of the glomerular area; score 4, sclerosis occupies 76–100% of the glomerular area. These scores were used to calculate GSI using the formula:


$$\begin{array}{l} GSI=\left(1xN1+2xN2+3xN3+4xN4\right)\\ \;/\left(N0+N1+N2+N3+N4\right) \end{array}$$

where *N* is the number of glomeruli with each grade of sclerosis. All glomeruli with GSI score 4 presented with global sclerosis, thus these terms are used synonymously.

### Glomerular and podometric analyses

#### Estimation of glomerular volume

**Figure 3:**
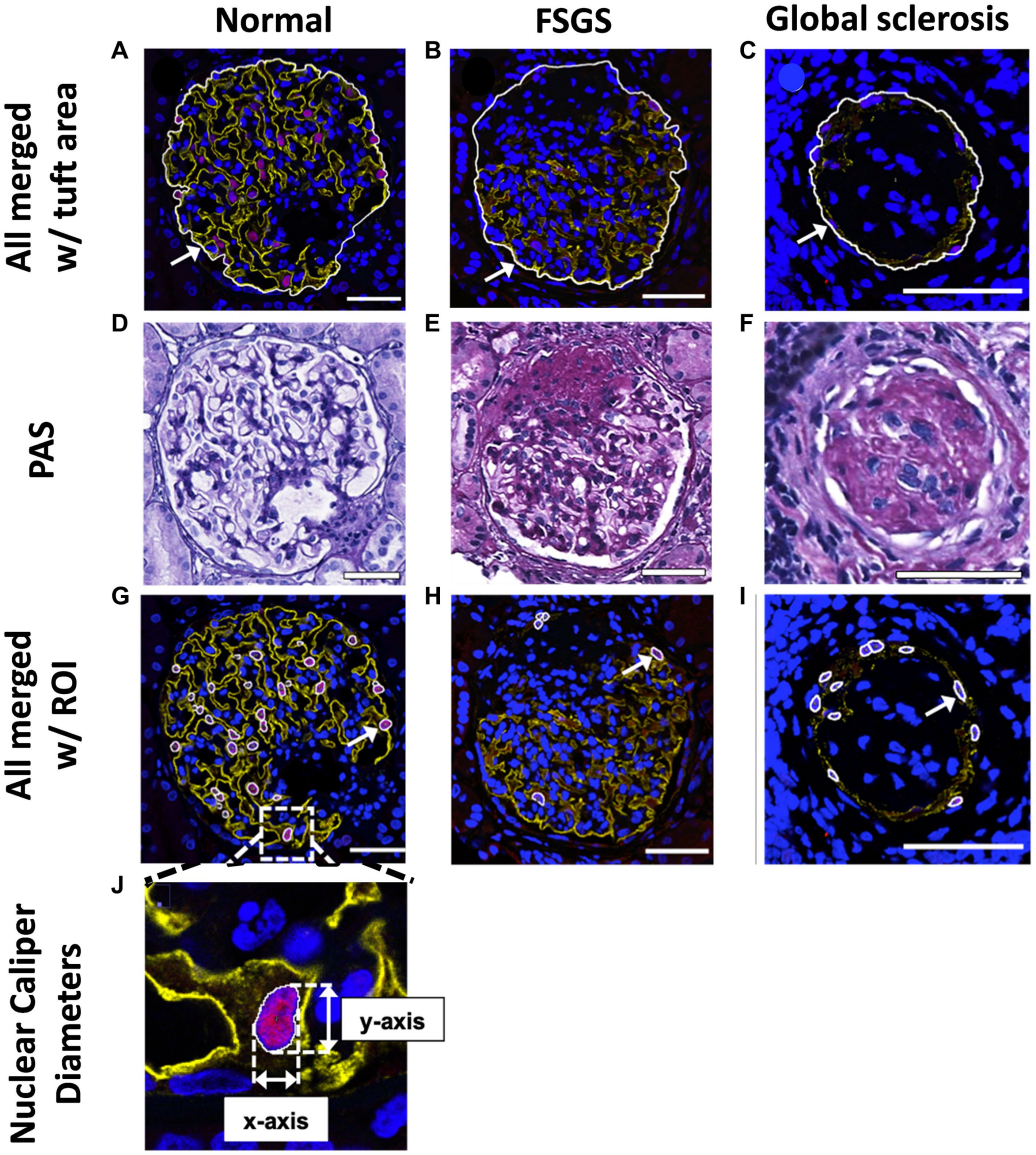
Glomerular tuft area and podocyte nuclear analysis. Mean glomerular area for each biopsy was determined and used to calculate mean glomerular volume. Glomerular tuft area (indicated with an arrow) was defined as the area of tissue immunofluorescently stained for podocyte cytoplasm and podocyte nuclei shown in a normal glomerulus **(A)**, a glomerulus with FSGS **(B)** and a glomerulus with global sclerosis **(C)**. Based on PAS images **(D–F)**, regions with glomerulosclerosis were included in the glomerular area analysis. **(G–I)**, DACH1 and DAPI merged images used to identify podocyte nuclei (DACH1^+^ and DAPI^+^) as regions of interest (ROIs), as indicated by arrows. Podocyte nuclear caliper diameters on the *x*- and *y*-axis were measured **(J)** and averaged for each biopsy. Scale bars = 50 μm.

All included patients had biopsies analyzed for podometrics based on the method of Venkatareddy et al. (39) modified by Haruhara et al. (45). Image files obtained using the SP5 confocal microscope were assessed for all podometric analyses using FIJI^®^ imaging software. Glomerular tuft area was assessed on merged immunofluorescent images of DACH1, synaptopodin and DAPI (Figures 3A–C) and defined as the area on the outer side of the capillary loops of the glomerular tuft. For glomeruli with sclerosis, the sclerotic region was also included in glomerular tuft area, where background staining and PAS images were used as a guide to estimate tuft area (Figures 3D–F). Mean glomerular area for each biopsy was calculated by averaging the measured areas of all glomerular tufts (46). Mean glomerular area was then used to calculate mean glomerular volume using the following Weibel and Gomez equation:


$$\mathrm{Glomerular}\;\mathrm{volume}=\beta /d\times {\left(\mathrm{mean}\;\mathrm{glomerular}\;\mathrm{area}\right)}^{3/2}$$

where β is a dimensionless shape coefficient (1.382 for spheres), and d is a size distribution coefficient used to adjust for variations in glomerular size. This study used a value of *d* = 1.01 (47, 48).

#### Estimation of podocyte number and density

Podocyte nuclei were defined as DACH1^+^ and DAPI^+^ and located outside glomerular capillaries, but not located on the Bowman’s capsule (Figures 3G–I). DACH1^+^ and DAPI^+^ nuclei located on Bowman’s capsule were considered parietal epithelial cells (42). Merged DAPI and DACH1 images were assessed using FIJI^®^ software, and DACH1^+^ and DAPI^+^ nuclei were marked as regions of interest (ROIs; Figure 3J). The XY-axis caliper diameters of all identified podocyte nuclei were measured and averaged for each biopsy (Figure 3J).

Podocyte density was calculated using the number of podocyte nuclei per biopsy, apparent podocyte nuclear caliper diameter, total glomerular area, and optical section thickness. Optical section thickness was calculated as 0.541 μm using the formula for axial resolution on confocal microscopy:


Axialresolution=0.88×λ/n−√n^2−NA^2

where λ is the excitation wavelength for DAPI (405 nm), n is the refractive index of the immersion oil (1.515), and NA is the numerical aperture of the objective lens (1.25). Podocyte number per tuft was calculated by multiplying podocyte density by glomerular volume.

#### Estimation of podocyte nuclear, cytoplasmic and total volumes

Podocyte cytoplasm was defined as synaptopodin^+^. A binary image of the synaptopodin file obtained from the confocal microscope was created using the IsoData algorithm (49) and used to measure the synaptopodin^+^ area within each glomerulus. The percentage of glomerular area with synaptopodin immunostaining was defined as the sum of all synaptopodin^+^ areas in glomerular tufts divided by the sum of all glomerular tuft areas in the biopsy section. The average volume of cytoplasm per podocyte was calculated as follows:


$$\begin{array}{l} \mathrm{Podocyte}\;\mathrm{cytoplasmic}\;\mathrm{volume}=\left(\begin{array}{l} \mathrm{Glomerular}\;\mathrm{volume}\\ \times \left(\%\mathrm{Synaptopodin}\right) \end{array}\right)\\ \;/\left(\mathrm{Podocyte}\;\mathrm{number}\;\mathrm{per}\;\mathrm{tuft}\right) \end{array}$$

Average podocyte nuclear volume was estimated based on the mean apparent caliper diameter of podocyte nuclei using the following formula (50, 51):


$$\mathrm{Podocyte}\;\mathrm{nuclear}\;\mathrm{volume}=4\pi /3\times \left(\begin{array}{l} 2/\pi \times \mathrm{apparent}\;\mathrm{caliper}\\ \mathrm{diameter}\;\mathrm{of}\;\mathrm{podocyte}\;\mathrm{nuclei} \end{array}\right)$$

Average podocyte volume was calculated as the sum of podocyte cytoplasmic volume and podocyte nuclear volume (52). Podocyte volumetric density in glomeruli (V_VPod/Glom_) was defined as the proportion of glomerular tuft volume comprised by podocytes, and was estimated using:


$$\begin{array}{l} V_{V}\left(\mathrm{Pod}/\mathrm{Glom}\right)=\left(\begin{array}{l} \mathrm{Podocyte}\;\mathrm{volume}\\ \times \mathrm{podocyte}\;\mathrm{number}\;\mathrm{per}\;\mathrm{tuft} \end{array}\right)\\ \;/\left(\mathrm{Glomerular}\;\mathrm{volume}\right) \end{array}$$

### Statistical analysis

Data were analyzed using GraphPad Prism software (Version 8, GraphPad Software, Inc., USA). Continuous data were tested for normality using the D’Agostino-Pearson omnibus normality test, and depending on the outcomes data were analyzed using an un-paired *t*-test (parametric data) or a Mann–Whitney test (non-parametric). Categorical data and difference between proportions were analyzed by Chi-Squared test. Most data were non-parametric and are therefore presented as median [inter-quartile range (IQR)]. Correlations between podometrics, GSI and urinary/serum data were analyzed with Spearman’s correlation test. Data are presented as trend-line ±95% confidence intervals (CI). A *p* value <0.05 was considered statistically significant.

## Results

### Patient clinical characteristics

#### Patient demographics and kidney function at time of biopsy

Patient demographics in the treatment responder and non-responder groups were similar (Table 1), with the exception of sex, with all six non-responders being male. Treatment responders and non-responders had similar levels of proteinuria at the time of biopsy, with median proteinuria in both groups >3.5 g/d (Table 1). Kidney function at the time of biopsy was also similar in the two groups, with median eGFR in both groups categorized as G2 GFR for CKD, which KDIGO defines as mildly decreased kidney function.

**Table 1:** Patient demographics and urine/serum data at time of biopsy.

	Responders (13)	Non-responders (6)	*p*
Demographics			
Age, years	35 (23–53)	45 (41–58)	0.16
Sex; male, n (%)	7 (56%)	6 (100%)	<0.05
Ethnicity; Caucasian, n (%)	11 (85%)	5 (83%)	0.94
Hypertension, n (%)	4 (31%)	2 (33%)	0.91
Smoking, n (%)	1 (8%)	2 (33%)	0.15
Urine/serum data at time of biopsy			
Proteinuria, g/24 h	3.87 (2.14–6.75)	5.83 (5.18–6.77)	0.08
Serum creatinine, μmol/l	88 (65–114)	116 (84–161)	0.13
eGFR*, ml/min/1.73 m^2^	84 (62–118)	62 (51–94)	0.22

Continuous data are median (IQR). *Indicates one patient from each group was <18 years of age, for which eGFR was not calculated.

#### Patient biopsy, treatment and response

38% of patients who responded to treatment were prescribed steroids only, and another 38% were prescribed steroids and medications to regulate blood pressure (Table 2). Notably, 23% of treatment responders were only prescribed blood pressure medication. Of the patients who did not respond to treatment, 17% were prescribed steroids only, and 50% received steroids and ACEi/ARBs.

**Table 2:** Initial treatment regime and 6 month outcomes.

	Responders (13)	Non-responders (6)	*p*
Initial treatment regime			
Steroids only, n (%)	5 (38%)	1 (17%)	0.34
Steroids + ACEi/ARBs, n (%)	5 (38%)	3 (50%)	0.63
ACEi/ARBs only, n (%)	3 (23%)	2 (33%)	0.64
Urinary/serum data 6 months after treatment			
Proteinuria, g/24 h*	1.00 (0.20–1.90)	6.11 (3.92–9.14)	<0.001
Serum creatinine, μmol/l*	91 (74–107)	121 (92–227)	0.053
eGFR, ml/min/1.73 m^2^*	85 (62–120)	61 (41–86)	0.14

Continuous data are median (IQR). *Indicates incomplete dataset (see Methods for justification).

As expected, non-responders had a higher level of proteinuria 6 months after diagnosis than responders (*p* < 0.001; Table 2). There was a trend toward lower eGFR in non-responders at 6 months, but this difference was not statistically significant (*p* = 0.14).

**Figure 4:**
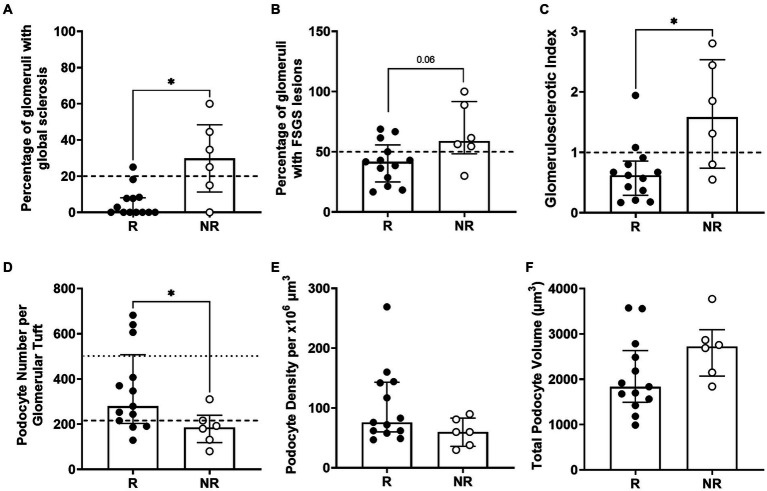
Biopsy analysis of glomerulosclerosis and podometrics. Percentage of glomeruli with global sclerosis, with the potential threshold of 20% global sclerosis (dashed line) as an indicator of treatment response **(A)**. Percentage of glomeruli with FSGS in responder and non-responder patients with a threshold of 50% of glomeruli with FSGS lesions (dashed line) as an indicator of treatment response **(B)**. Glomerulosclerotic index in responder and non-responder patients with a threshold GSI of 1 (dashed line) to indicate response to treatment **(C)**. Podocyte number per glomerular tuft, where the average normal value of ~500 podocytes per glomerulus in healthy adults is indicated by a dotted line and the dashed line at 216 podocytes indicates the potential threshold of podocytes/glomerulus that could identify treatment response **(D)**. Glomerular podocyte density **(E)** and podocyte volume per glomerulus **(F)** were similar in responders and non-responders. Each point represents a patient who responded to treatment (R; black; *n* = 13) or did not (NR; white; *n* = 6). Continuous data tested by Mann–Whitney test and proportion analysis tested with Chi-Square test. Data are median ± IQR. * indicates *p* < 0.05.

Patients who did not respond to treatment at 6 months had a significantly higher percentage of glomeruli with global sclerosis than responders at diagnosis (*p* < 0.05; Figure 4A). A greater percentage of glomeruli in non-responders had FSGS lesions although this did not reach statistical significance (*p* = 0.06; Figure 4B). Non-responders had a significantly higher GSI than responders (*p* < 0.05; Figure 4C). Importantly, 67% of non-responders (4/6) compared to only 7% of responders (1/13) had greater than 20% of glomeruli with global sclerosis (*p* < 0.01; Figure 4A). Similarly, 83% of non-responders (5/6) compared to only 30% of responders had greater than 50% of glomeruli affected by FSGS lesions (*p* < 0.05; Figure 4B) and 67% of non-responders (4/6) compared to 15% of responders (2/13) had a GSI greater than 1 (*p* < 0.05; Figure 4C). Together, these findings indicate a threshold of 20% global sclerosis, 50% of glomeruli with FSGS lesions and a GSI of 1 or more may indicate the response to first line therapy (Figures 4A–C).

### Podometric analyses

Median podocyte number per glomerulus in treatment responders was 279 (203–507), 50% greater than that of non-responders (186, 118–310; *p* < 0.05; Table 3; Figure 4D). Interestingly, the proportion of patients with a podocyte count of 216 or less was significantly higher in non-responders (83%) than responders (30%), indicating podocyte number per glomerulus can also indicate the response to first line therapy (*p* < 0.05; Figure 4D). Glomerular volume was similar in the two groups. Podocyte density was 27% greater in responders than non-responders, but this difference was not statistically significant (*p* = 0.12; Table 3; Figure 4E).

**Table 3:** Podometric data.

Variable	Responders (13)	Non-responders (6)	*p*
Analysis parameters			
Number of glomerular profiles evaluated/biopsy	13 (10–16)	13 (9–20)	0.99
Apparent caliper diameter of podocyte nuclei, μm	7.31 (7.11–7.70)	7.64 (6.90–8.02)	0.57
Estimated true diameter of podocyte nuclei, μm	9.95 (9.67–10.50)	10.4 (9.39–10.90)	0.57
Podocyte number and density, and glomerular volume			
Podocyte number per glomerular tuft	279 (203–507)	186 (118–310)	<0.05
Glomerular volume, x10 μm^3^	3.88 (2.76–4.56)	2.95 (2.35–4.85)	0.64
Podocyte density, per 10^6^ μm^3^	76 (60–143)	60 (36–83)	0.12
Podocyte volume indices			
% of glomerulus stained for SNP	13 (11–19)	12 (9–18)	0.37
Podocyte nuclear volume, μm^3^	399 (357–452)	456 (295–530)	0.37
Podocyte cytoplasmic volume, μm^3^	1,425 (1122–2,167)	2,286 (1627–2,549)	0.07
Total podocyte volume, μm^3^	1834 (1493–2,632)	2,721 (2072–3,092)	0.08
Podocyte nuclear to cytoplasmic ratio	0.25 (0.21–0.37)	0.22 (0.19–0.25)	0.11
V_V(Pod/Glom)_, %	17 (15–23)	15 (10–22)	0.37

Data are median (IQR). V_V(Pod/Glom)_: percentage of glomerular volume comprised by podocytes.

The proportion of glomerular volume comprised by podocyte cytoplasm was similar in responders and non-responders (Table 3). Podocyte volume was 48% greater in non-responders (2,721, 2,072–3,092 × 10 μm^3^) than responders (1,834, 1,493–2,632 × 10 μm^3^), however this difference was not statistically significant (*p* = 0.08; Table 3; Figure 4F).

#### Remaining podocytes increase in size following absolute and relative podocyte depletion

**Figure 5:**
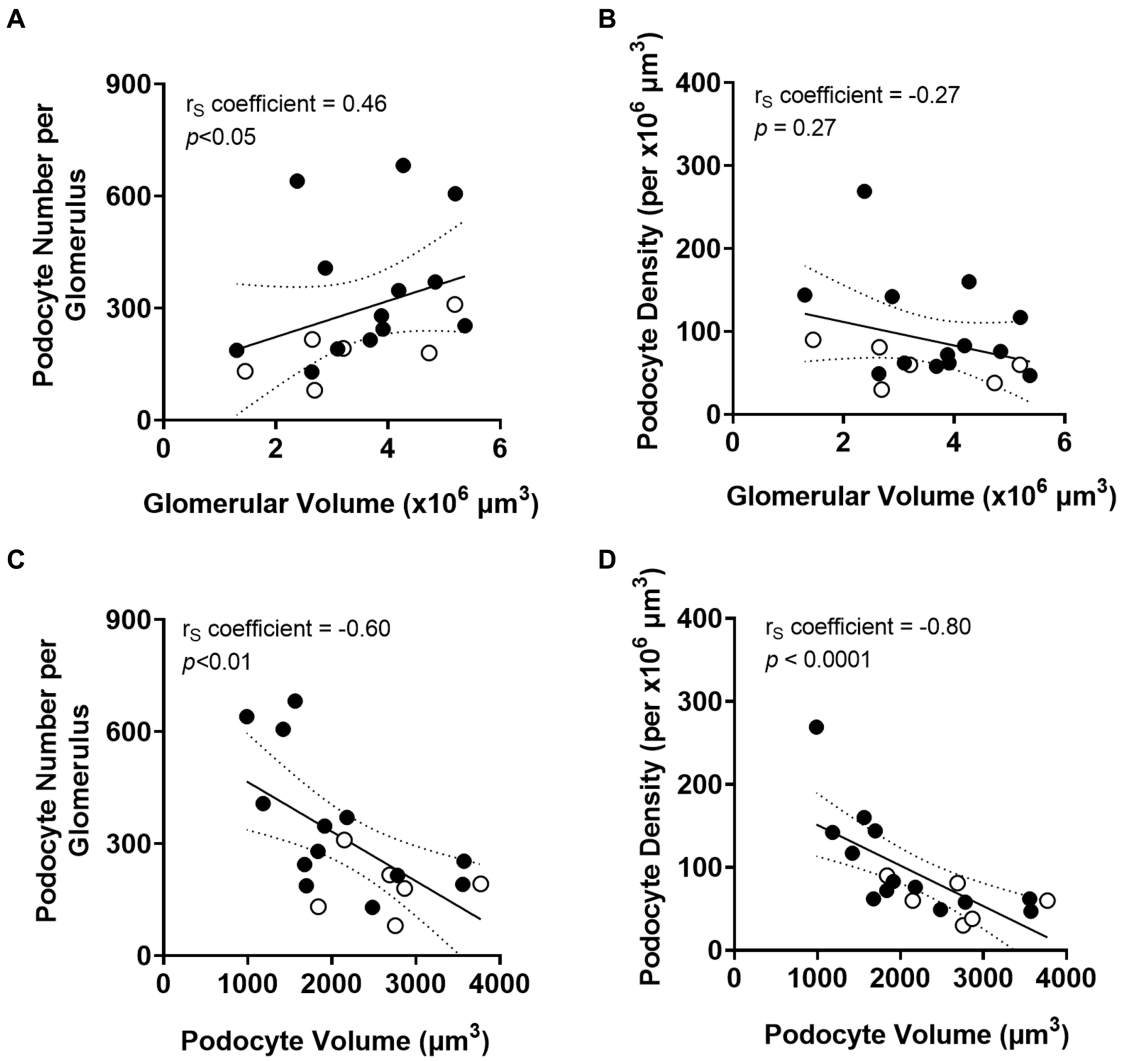
Correlations between podometrics and glomerular size. Correlation analyses between glomerular volume and podocyte number **(A)**, and between glomerular volume and podocyte density **(B)**. Correlations between podocyte volume and podocyte number **(C)**, and between podocyte volume and podocyte density **(D)**. Trend line ±95% CI. Responders (black), non-responders (white).

Total podocyte number per glomerulus correlated directly with glomerular volume (*p* < 0.05; Figure 5A). However, there was no relationship between podocyte density and glomerular volume (Figure 5B). Average podocyte volume negatively correlated with podocyte number (*p* < 0.01; Figure 5C), suggesting that as podocyte number decreases, remaining podocytes undergo hypertrophy. Average podocyte volume was inversely correlated with podocyte density (*p* < 0.0001; Figure 5D).

#### Podocyte depletion is a key event in the development of glomerulosclerosis

**Figure 6:**
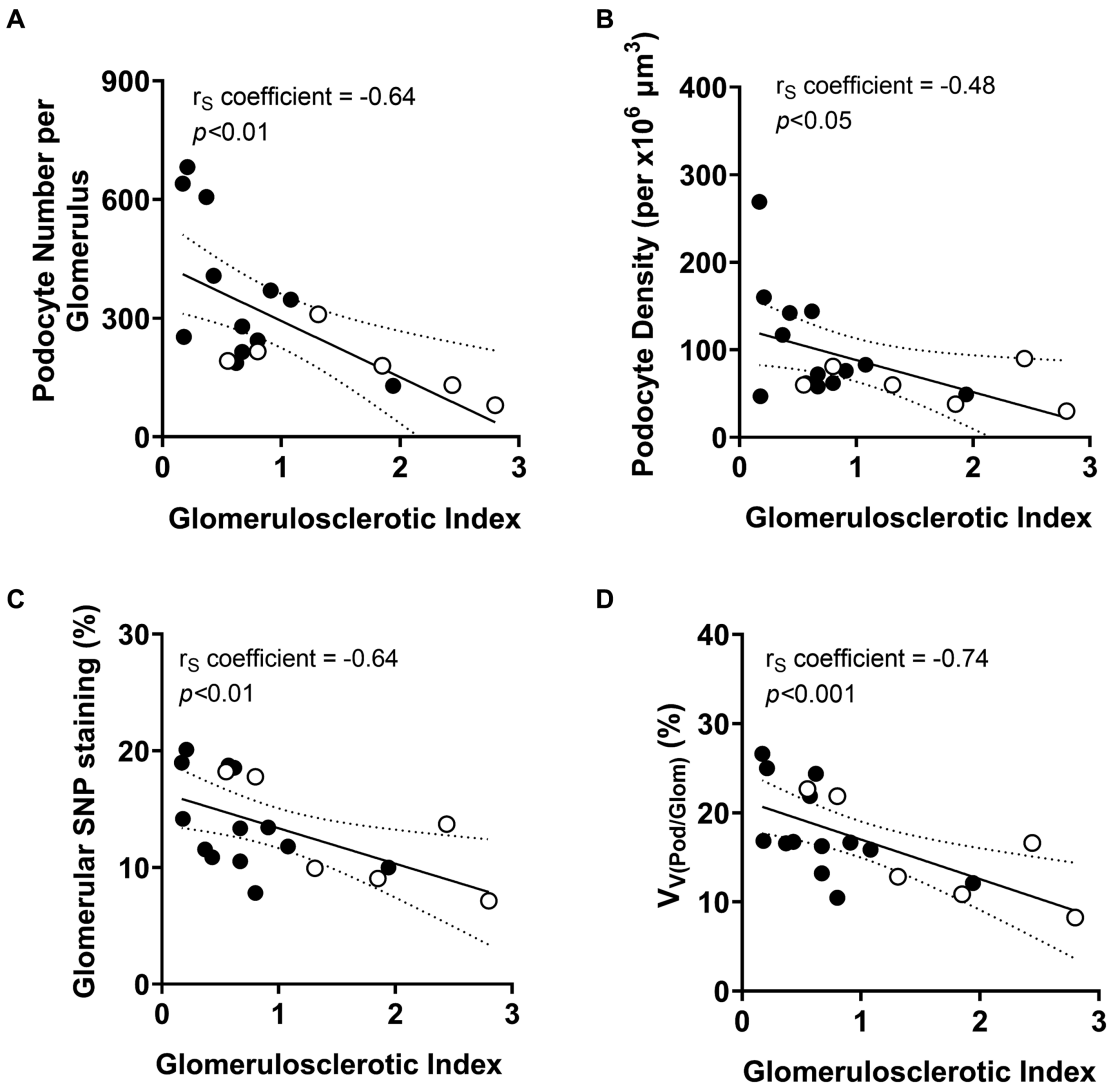
Correlations between podometric parameters and glomerulosclerosis. Correlation analyses between GSI and **(A)** podocyte number, **(B)** podocyte density, **(C)** glomerular synaptopodin (SNP) staining and **(D)** percentage of glomerular volume comprised by podocytes (V_V(Pod/Glom)_). Trend line ±95% CI. Responders (black), non-responders (white).

As expected, GSI at the time of diagnostic biopsy was negatively correlated with both podocyte number (*p* < 0.01; Figure 6A) and podocyte density (*p* < 0.05; Figure 6B). The extent of glomerulosclerosis negatively correlated with the percentage of glomerular volume constituted by podocyte cytoplasm (*p* < 0.01; Figure 6C). The percentage of glomerular volume comprised by podocytes was also negatively correlated with severity of glomerulosclerosis (*p* < 0.001; Figure 6D).

#### Podometrics and urinary/serum data

**Figure 7:**
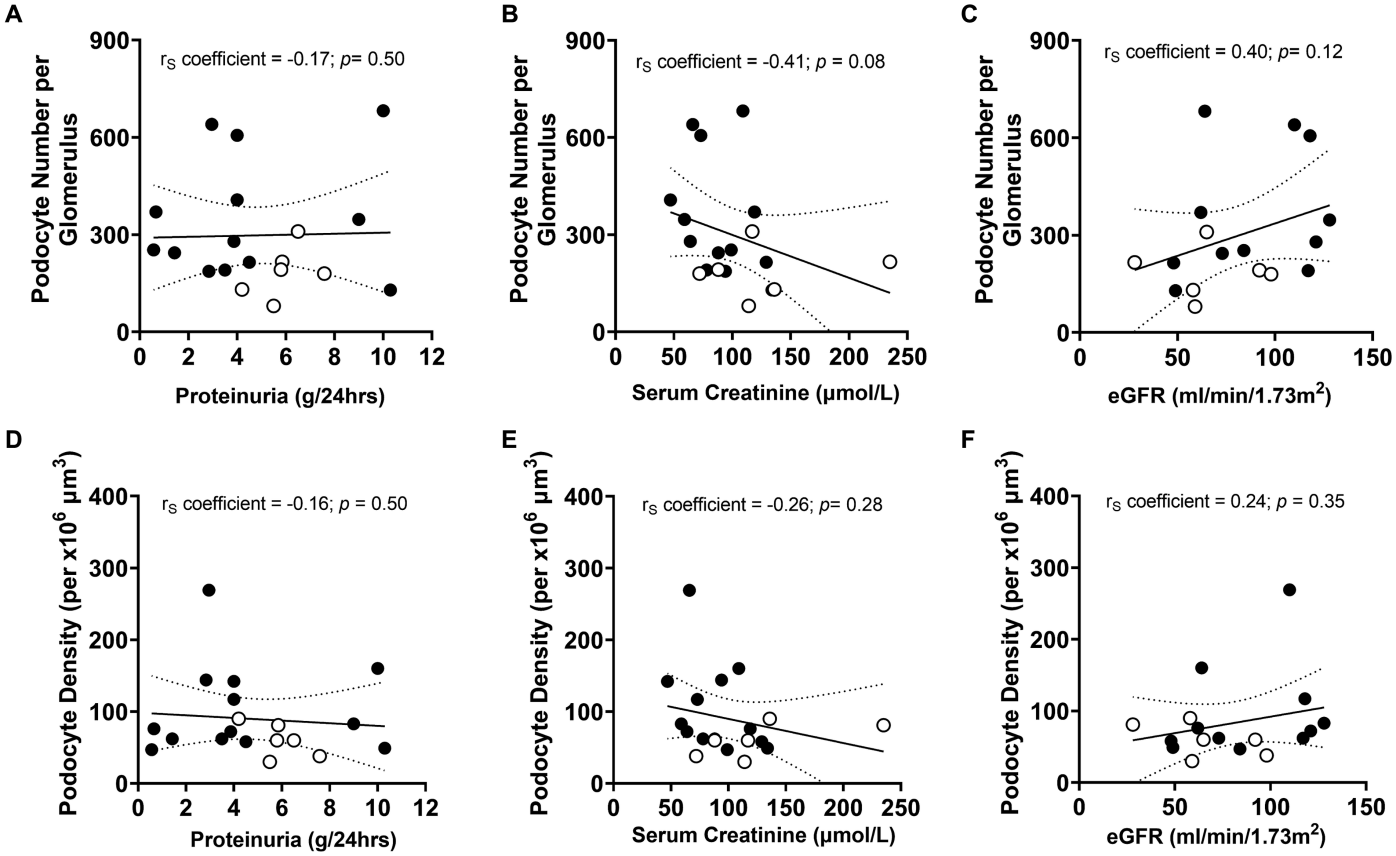
Correlations between podometric and urinary/serum data at biopsy. Correlations between podocyte number and **(A)** proteinuria, **(B)** serum creatinine and **(C)** eGFR. Correlations between podocyte density and **(D)** proteinuria, **(E)** serum creatinine and **(F)** eGFR. Trend line ±95% CI. Responders (black), non-responders (white).

Given podocyte number was significantly lower in non-responders than responders, the relationships between podocyte number per glomerulus and proteinuria, serum creatinine and eGFR at biopsy were investigated to determine if non-invasive urinalysis could be used to determine the severity of podocyte depletion at diagnosis (Figures 7A–C). A negative correlation between podocyte number and serum creatinine at biopsy approached statistical significance (*p* = 0.08; Figure 7B). However, no other associations between other urinary/serum data at biopsy and podometrics were observed. Similarly, there were no statistical relationships between podocyte density and proteinuria, serum creatinine or eGFR (Figures 7D–F).

## Discussion

This is the first study to analyze podometrics and indices of glomerulosclerosis in diagnostic biopsies of patients with primary FSGS who were subsequently shown at 6 months to be either responders or non-responders to first-line therapy. Our results show that total podocyte number per glomerulus at the time of biopsy was significantly higher in responders than non-responders at 6 months, whereas glomerulosclerotic index and the percentage of glomeruli with global sclerosis were significantly lower in responders. Moreover, threshold values at the time of biopsy of approximately 216 podocytes per glomerulus, 20% of glomeruli with global sclerosis, 50% of glomeruli with FSGS lesions and a GSI score of 1 were able to indicate the response to first line therapy at 6 months.

The mechanisms of action and target cells of corticosteroids are still not completely understood, although recent studies suggest the efficacy of glucocorticoid therapy is achieved, at least in part, through direct effects on podocytes (53). The present findings demonstrate that patients with primary FSGS who responded to first-line therapy had more podocytes per glomerulus at the time of biopsy than patients who did not respond. Studies have also reported increased podocyte survival and preservation of podocyte number following corticosteroid therapy (24, 54).

Although steroid treatment is considered first-line therapy in primary FSGS, it is avoided in patients in whom side effects of steroids are of particular concern (22). For these patients, more conservative therapy may be considered such as blood pressure control achieved with RAS blockade (ACEi/ARBs) and dietary modifications (8), as observed in approximately 30% of patients in both groups in the present study. Moreover, steroid treatment is often prescribed in combination with ACEi/ARBs, which was the case in 38% of responders and 50% of non-responders in the present study. RAS blockade lowers systemic blood pressure which in turn reduces intraglomerular capillary pressure, reducing mechanical distension (stress) of the capillary tuft and thereby protecting podocytes (55). However, podocytes express angiotensin II type 1 receptors and this increases following mechanical stress *in vitro* (56). Mechanical stress on podocytes also increased podocyte angiotensin II production and apoptosis by 2.5 fold, which was ameliorated by RAS blockade (56). Taken together, these findings suggest that ACEi/ARBs have direct effects on podocyte survival, independent from systemic blood pressure control.

Normal adult human glomeruli contain approximately 500–600 podocytes each (57, 58). Interestingly, several studies have identified that a podocyte number of approximately 200 or less can indicate poorer outcomes. Lemley et al. (59) found that patients with IgA nephropathy and with fewer than 200 podocytes per glomerulus had increased frequency of GFR < 90 mL/min/1.73 m^2^, a higher percentage of global glomerulosclerosis and high shunt magnitude. Similarly, Andeen et al. (60) found patients with early stage diabetic nephropathy had 268 ± 73 (mean ± standard deviation) podocytes per glomerulus, whilst those with advanced disease had 144 ± 52 podocytes per glomerulus. Although these studies did not evaluate therapeutics specifically, the findings support the concept that patients with podocytopathies with fewer than around 200 podocytes per glomerulus are of increased clinical concern and may require more intricate therapeutic strategies than standard first-line therapy. Similarly, ten of the nineteen patients in the present study had 216 or fewer podocytes per glomerulus - this included five of the six non-responders and four of the thirteen responders, suggesting that patients with fewer than approximately 200 podocytes per glomerulus may be more unlikely to respond to first-line therapy.

In contrast to podocyte number per glomerulus, podocyte density is an index of relative podocyte depletion which has repeatedly been shown to be a key event in the onset and progression of glomerulosclerosis (61, 62). We therefore expected that differences in both podocyte number and podocyte density would be found between responders and non-responders to first-line therapy. However, we found no difference in podocyte density between these two groups.

Although the present study found that podocyte density may not be useful in identifying patients who are likely to respond to first-line therapy, podocyte density undoubtedly remains a very valuable tool for assessing glomerular disease. A podocyte density of 100 podocytes per 10^6^ μm^3^ glomerular volume is considered a critical threshold that results in dysfunctional glomeruli, protein leakage, glomerulosclerosis and decreased renal function (63). Further decreases in podocyte density require exponential increases in podocyte hypertrophic compensatory adaptations. However, this is difficult to accomplish given that podocytes have a limited capacity to increase in size. Failure of podocyte hypertrophy to match the enlarged glomerulus drives progression of glomerular disease (64).

Median podocyte density for both groups in the present study was less than 100 per 10^6^ μm^3^ at the time of biopsy. Interestingly, all non-responders had a podocyte density less than this value, as did 62% of responders. For both groups of patients, podocyte volume was significantly and negatively correlated with podocyte number and podocyte density, evidence of podocyte hypertrophy. Although not statistically significant, it is of interest that podocyte cytoplasmic volume was 60% higher in non-responders than responders (*p* = 0.07), while total podocyte volume was 48% higher in non-responders (*p* = 0.08). This suggests that podocytes in non-responders had undergone greater hypertrophy than podocytes in responders, indicative of increased podocyte hypertrophic stress. Non-responders also had a higher level of glomerulosclerosis. Taken together, these data support the podocyte depletion hypothesis (6, 64).

In addition to podocyte number being able to distinguish treatment responders from non-responders in the present study, three indices of glomerulosclerosis also distinguished between these patients – the percentage of glomeruli with global sclerosis, the percentage of glomeruli with FSGS lesions, and GSI. While these findings are perhaps not surprising, to our knowledge this is the first study to report that indices of glomerulosclerosis have predictive clinical value in patients with primary FSGS. Interestingly, in a study of ANCA-associated glomerulonephritis, Brix et al. (65) developed and validated a clinicopathologic score to indicate renal outcomes. They found that the percentage of normal glomeruli (without scarring, crescents or necrosis) was the strongest indicator of death-censored end stage renal disease. Brix et al. (65) also identified predictive threshold values for these three parameters. With the recent development of deep learning algorithms and new morphometrics to interrogate renal histopathology (66), one can envisage rapid progress in the development of new strategies to indicate kidney outcomes from diagnostic biopsies and functional data and thereby more accurately indicate and refine therapy.

The relationships between urinary/serum data at biopsy and podocyte number per glomerulus and podocyte density were analyzed to determine if non-invasive urinalysis could be used to determine the severity of podocyte depletion at diagnosis. No significant correlations were observed, although the relationship between serum creatinine and podocyte number approached statistical significance. It was expected that increased proteinuria and serum creatinine, and decreased eGFR would be associated with greater podocyte depletion. This was the case in a study of patients with IgA nephropathy where podocyte density was significantly correlated with serum creatinine and eGFR (67).

A limitation of this single center study is the relatively small sample size. Archival kidney biopsy tissue was obtained from patients who required a biopsy to confirm diagnosis of primary FSGS over a 12-year period (2009 to 2020). While 84 patients were initially recruited, 65 patients were excluded, leaving 13 responders and 6 non-responders. While novel and clinically relevant findings were obtained, a larger sample size may have found that podocyte density was higher in responders than non-responders, as was found for podocyte number. The small study size also meant that differences between groups could not be fully appreciated. For instance, non-responders were older, but the age difference between groups was not significant. Treatment regimens also differed, with not all study patients having documented ACEI/ARB therapy which is now standard of care alongside any immunosuppressive therapy (8, 9). Due to the retrospective nature of the study, data collection was limited to what was recorded in patient notes whereby 24-h urine protein results were not available for 16% of patients at time of biopsy and 74% of patients at 6 months. However, spot urine protein levels have been shown to be comparable to 24 h urine protein calculations for the clinical outcomes of end stage renal failure and death (68). Given the retrospective nature of the study and the small sample size, further studies incorporating stratification for confounders such as patient age and treatment regimen are required to fully understand the utility of podometrics and glomerulosclerosis indices for determining therapeutic options.

In conclusion, these findings suggest that primary FSGS patients with higher podocyte number per glomerulus and less glomerulosclerosis at the time of diagnostic biopsy respond to first-line therapy at 6 months. A podocyte number of less than approximately 200 per glomerulus, a GSI greater than 1, and a percentage global sclerosis greater than 20% may all be associated with a lack of response to first-line therapy. Podometric and glomerulosclerosis analyses of diagnostic biopsies may be of clinical value in determining optimum therapeutic options for patients with primary FSGS, however, larger, prospective studies would be needed to confirm this.

## Data availability statement

The raw data supporting the conclusions of this article will be made available by the authors, without undue reservation.

## Ethics statement

The studies involving humans were approved by the Monash University Human Research Ethics Committee (14344). The studies were conducted in accordance with the local legislation and institutional requirements. Written informed consent for participation in this study was provided by the participants’ or their legal guardians/next of kin.

## Author contributions

NZ: Data curation, Formal analysis, Investigation, Writing – original draft. KH: Methodology, Writing – review & editing. DN-P: Methodology, Supervision, Writing – review & editing. PK: Investigation, Methodology, Supervision, Writing – review & editing. JL: Data curation, Investigation, Methodology, Writing – review & editing. SG: Methodology, Writing – review & editing. VP: Conceptualization, Writing – review & editing. JB: Conceptualization, Project administration, Supervision, Writing – review & editing. LC-M: Conceptualization, Data curation, Formal analysis, Project administration, Supervision, Writing – original draft, Writing – review & editing.
